# Transcending toward Advanced 3D-Cell Culture Modalities: A Review about an Emerging Paradigm in Translational Oncology

**DOI:** 10.3390/cells10071657

**Published:** 2021-07-01

**Authors:** Joviana Farhat, Ishan Pandey, Mohammad AlWahsh

**Affiliations:** 1 College of Pharmacy, Al Ain University, Abu Dhabi 112612, United Arab Emirates; joviana.w.farhat@gmail.com; 2 Department of Pathology, Motilal Nehru Medical College, Prayagraj 211001, India; ishan.pandey411@gmail.com; 3 Leibniz-Institut für Analytische Wissenschaften-ISAS-e.V., 44139 Dortmund, Germany; 4 Institute of Pathology and Medical Research Center (ZMF), University Medical Center Mannheim, Heidelberg University, 68167 Mannheim, Germany; 5 Department of Pharmacy, Faculty of Pharmacy, Al-Zaytoonah University of Jordan, P.O. Box 130, Amman 11733, Jordan

**Keywords:** cancer, 3D-cell culture, 2D-cell culture, cellular microenvironment activity

## Abstract

Cancer is a disorder characterized by an uncontrollable overgrowth and a fast-moving spread of cells from a localized tissue to multiple organs of the body, reaching a metastatic state. Throughout years, complexity of cancer progression and invasion, high prevalence and incidence, as well as the high rise in treatment failure cases leading to a poor patient prognosis accounted for continuous experimental investigations on animals and cellular models, mainly with 2D- and 3D-cell culture. Nowadays, these research models are considered a main asset to reflect the physiological events in many cancer types in terms of cellular characteristics and features, replication and metastatic mechanisms, metabolic pathways, biomarkers expression, and chemotherapeutic agent resistance. In practice, based on research perspective and hypothesis, scientists aim to choose the best model to approach their understanding and to prove their hypothesis. Recently, 3D-cell models are seen to be highly incorporated as a crucial tool for reflecting the true cancer cell microenvironment in pharmacokinetic and pharmacodynamics studies, in addition to the intensity of anticancer drug response in pharmacogenomics trials. Hence, in this review, we shed light on the unique characteristics of 3D cells favoring its promising usage through a comparative approach with other research models, specifically 2D-cell culture. Plus, we will discuss the importance of 3D models as a direct reflector of the intrinsic cancer cell environment with the newest multiple methods and types available for 3D-cells implementation.

## 1. Introduction

There are several cancer model systems available for studying the disease pathways and screening therapies. Although all of the models have contributed valuable knowledge about cancer biology, the new methods have major flaws. Approximately 90% of potential preclinical medications in all therapeutic groups fail to result in effective human treatments, wasting large amounts of time and resources and, ultimately, delaying the discovery of successful interventions [1]. Two-dimensional (2D) tissue culture models, in the most simplified perspective, lack realistic sophistication, whereas animal models are costly, time consuming, and due to ethical concerns it would be great to reduce it [2]. When designing cancer models for preclinical screening and monitoring, as well as new therapy research and development, adding 3D culture to the laboratory’s arsenal will accelerate discovery and save money.

To study the diverse components of tumor and their care, researchers have developed a wide range of model systems, each with its own set of advantages and drawbacks. There are two important conditions for all versions. The first being the ability to regulate cell number and viability, since unregulated cell proliferation is a central feature of cancer, which is maintained by various mechanisms [3]. The second requirement is the capability to research cancerous cell proliferation and infiltration of adjacent tissues, since metastasis not only affects surrounding tissues but also complicates the treatment. Angiogenesis enhancement and immune system evasion are two complex and beneficial but difficult-to-model characteristics that lead to long-term tumor survival. The ideal preclinical model will be relatively affordable, amenable to high-throughput screening, and most significantly, closely reflect human-tumor biology. Animal models have been used as living incubators for human tumors, allowing them to interact with an entire body, although often an immunocompromised one. Cancer is developed in rodent models by either surgically implanting tumor cells or developing genetically modified animals that produce human-like tumors spontaneously in response to experimental gene expression alteration [4].

At first sight, this seems to be an intrinsically beneficial system for simulating human illness. Despite widespread use for screening, these costly animal models have struggled to convert into treatments that improve human disease results [3,4,5]. Furthermore, there are many types of deadly cancer that actually lack a qualified animal model, such as brain, kidney, and skin cancer [5]. The causes of animal model failures are not well-known. Determining why this will lead to a better view of the disease mechanism in the long run, but for the time being, these models are simply not producing treatments with a high rate of return on investment.

Scientists have been working on 3D-tissue culture models to resolve the difference between in vitro studies used for exploration and sampling and in vivo experiments used for effectiveness and safety evaluation prior to moving forward with clinical trials. To dissect cancer biology and develop therapeutic screens, it is reasonable to use 3D models with personalized microenvironments, and proof that they are a superior method to 2D and early stage animal research is rapidly evolving [6].

Over the past decade, we have made enormous progress in our understanding of the anti-cancer immune system. These advancements range from fundamental to clinical science. The structures used in research biology must become more complicated as the biology discovered becomes more complex. These research needs have been met by 3D-culture models, which provide dynamic and interpretable platforms. So far, 3D-culture systems have been used to gain new insights into immunotherapy delivery, immune cell penetration, cancer-induced immunosuppression, CAR cell growth, and many other topics. Other cancer-immunology-related areas where 3D-culture systems are yet to be introduced include, but are not limited to, changes in regulatory T-cell migration and function, myeloid-derived-suppressor cell biology, and vaccine production. As the cost of 3D technology declines, availability increases, and conceptual implementations improve, these approaches will inevitably gain acceptance, perhaps supplanting 2D-culture methods entirely [7].

Orthodox two-dimensional (2D) monolayer cell culture systems are used in approximately 90% of in vitro studies. Owing to the failure of 2D-cell culture systems to reliably recapitulate the structure, activity, and physiology of living tissues, multiple experiments such as testing the effectiveness of novel therapies, studying gene expressions, metabolic processes, and cell proliferation do not correspond to real in vivo scenarios [8]. The main explanation for this is due to two factors: (1) in the natural world, cancer cells undergo complex limited diffusion of oxygen, nutrients, and signaling molecules, which the 2D struggles to mimic. (2) Cellular association, activity, development, and signaling all take place in a highly complex 3D architecture influenced by extracellular matrix and other regulatory factors that cannot be replicated in 2D systems [9].

Microfluidic cell culture systems, which can provide a constant supply of nutrients, gas exchange, and other regulating factors in a well-controlled manner, can be used to accomplish this complex synchronization. A device like this is suitable for simulating the in vivo state of cells. The combination of microfluidics and 3D-cell culture systems would allow the analysis of cellular functions such as proliferation in dynamic systems, cell–cell interaction, and cellular response to the external environment in a far more practical environment. Furthermore, microfluidic systems allow the analysis of cellular activity by fabricating microstructures and artificial scaffolds to investigate cellular movements and the underlying mechano-biology. Using the microfluidic approach, better drugs and therapies that can be conveniently converted to in vivo systems can be created, bridging the difference between in vitro and in vivo systems [10].

Up to date 2D in vitro experimental trials have been always the most widely used method for approaching any cell mechanism and its possible interactions [11], secretion of neurotransmitters and hormones, positive and negative feedback systems [12], pathways signaling [13]. Despite this remarkable progress, cancer-related tumor characteristics [14], disease progression as well as the mechanism of action of a newly discovered drug [15] or even the modification of an old drug [11] characteristics in order to improve patient’s quality of life [16], adherence, compliance, and death rates are still subject to various obstacles [17]. Nowadays, oncology clinical trials using experimental models confirmed the necessity of reflecting the in vivo tissues’ architecture [18], complete pathophysiology of a cancerous cell and its cell-extracellular matrix (ECM) interactions [19]. Despite their previous high implementation, the two dimensional cells have been associated with no or limited efficacy in reflection of real patients tumor cases [20]. Same for the animal models with their high costs, species variations, confined availability and feasibility with the crucial need for ethical approvals [21]. Along with the alarming increase in allele mutations [22], protein disruption and genetic expressions, 2D-tissue-based studies were gradually substituted by a 3D in vitro model imitating the more in vivo like physiologic architecture and microenvironment [23]. So, 3D-tumor cells were directly implicated as the primary bridge between the 2D cells and animal models in order to pre-clinically peer the in vivo cancer cells resistance mechanism toward any chemotherapeutic agent [24], radiotherapy or supportive treatment [25,26]. Thus, 3D-tumor models in vitro application provided major advances in research in terms of patient’s drug regimen improvement as well as a more accurate scientific hypotheses related to cancer [27]. Nowadays, a single 3D-cell line exposed to a uniform growth-promoting environment, have the intrinsic potential of functioning through an emergent and self-organized process that results in the formation of complex multicellular asymmetric structures. This newly developed 3D approach is based on multiple inclusion criteria favoring its usage as it is more close to reflective method toward any in vivo mechanism investigated by scientists [28]. Thus, in this review, we shed the light on the 3D technology implication in cancer science based on three main areas: cancer diagnostics, drug discovery, and next-generation therapies, as well as driving the attention toward all the promising advantages of 3D-tumor culture models application in cancer and its contribution toward an initial treatment success accompanied with minimal adverse events. In addition to the available techniques imposed for a well-structured 3D-cell culture and its present and future application settings.

## 2. Comparative Approach between 2D- and 3D-Cell Culture

Multiple recent studies reflected the advantages of using 3D-cell culture over the 2D-cell culture in function of cellular microenvironment activity, drug resistance, and the expression of intrinsic and genetic factors. In fact, 3D-culture systems can mimic the modeling of cellular microenvironment using the newly performed methods, especially in case of a disease state investigation. Thus, reducing the need for animal models [29]. Moreover, 3D models offer a more realistic approach for assessing a precise drug-dose response compared to a variable results obtained in 2D cells [30]. This is related to the presence of natural cellular barriers which allow a unique and smooth drug diffusion across multiple layer of cells not encountered in other models [31,32]. Moreover, scaffolds can strengthen 3D-cell growth in terms of growth factor, drug or gene delivery [33,34,35,36], highlighting its direct correlation with tissue engineering and regenerative conditions. Hence, 2D- and 3D-cell culture methods do share some common characteristics yet differential criteria remain dominant (Table 1).

## 3. Cancer Diagnostics

It involves the study of cancer tumor’s microenvironment (TME) and its (ECM) impact on the implementation of the ultra-modern 3D model types and their accompanying methods of preparation as scaffolds, organoids, and spheroids, as well as 3D technology integration in drug discovery through drug-dose response, intrinsic pathways/genetic factor’s expression assessment and microfluidics technique. In fact, all these developing 3D areas are considered the main factors in cancer diagnostics, granting a crucial promising value in immuno-oncology field.

### 3.1. Cellular Microenvironment

Throughout practical research journey, 3D-cell culture is classified as a main technique for further approaching the in vivo-like tumor microenvironment (TME). From one side, TME is composed of a diverse cellular milieu in which cancer stem cells (CSCs) multiplicate and mature. Mainly, stromal and immune cells are connected to construct and stabilize this self-sustained environment. Stromal and tumor cell crosstalk constitutes the base for the promotion of a well-organized TME, reaching effective immune evasion, ECM remodeling, and angiogenesis [48]. From the other side, (TME) acellular part is related to the physiological activation cycles, intermediate metabolites, and protective mechanisms. A recent clinical trial highlighted the proangiogenic effect post-preconditioning human mesenchymal stem cells (hMSCs) for 96 h on a three-dimensional (3D) ECM-based microgel platform. This effect drove (hMSCs) toward changes in extracellular stiffness and “outside-in” integrin signaling [49]. Inflammatory response was also alleviated through newly formed blood vessels. In fact, these 3D-promising mechanisms limited the progression of tissue damage along with a faster tissue repair and reperfusion, especially in “no-option” patients suffering from peripheral arterial diseases [50]. Moreover, self-organization and differentiation ability of pluripotent stem cells in vitro in three-dimensional aggregates, known as organoids or organ spheroids, reflected the human brain development and functioning [51]. Hence, proving that (TME) is not only based on cellular systems, intracellular/intercellular interactions, but also on the intrinsic environmental interactions like cytokines and growth factors release as well as the biochemical conditions which play a major role in mastering in vitro tumor modeling, thus reflecting the in vivo tumor conditions.

### 3.2. Extracellular Matrix (ECM)

The extracellular matrix (ECM) constitutes the widest component of the TME and is made of proteins such as collagen, proteoglycans, hyaluronic acid, and laminins [52]. The ECM plays an important role in TME maintenance and metastasis induction. Plus it is responsible for cellular adhesion and migration out of the TME. It is able to store angiogenic factors and chemokines responsible for continuous inflammatory state causing an expansion in the cellular repertoire [53]. Additionally, ECM affects the recruitment of immune cells into the TME. PI3K/AKT (pro-survival pathway) activation [54,55,56], activations of immunosuppressive cells such as Tregs (regulatory T cells) and TAMs (tumor-associated macrophages) promoting (CSC) survival, blocking anti-tumorigenic immune cell recruitment [57], impairing the proliferation and activation of T cells [57] are all a crucial (ECM) mechanism of actions. Consequently, ECM composition also plays a crucial role in stabilizing the state of tumor infiltrating immune cells [58,59]. In addition to the afore mentioned, neutrophils and TAMs are capable of uniquely detecting the EMC in order to initiate cancer growth as they are recruited to the microenvironment [60,61]. This implies the ability of the ECM to modulate immune surveillance in the CSC microenvironment and account for a prior target for 3D models’ site of action.

### 3.3. Ultra-Modern 3D Model Types and Methods of Preparation

Over years, 3D-cell culture research models are constantly improving in terms of multiple available methods for preparation based on scientists’ need and subject of investigation. As a result, these diverse 3D-cell types attained the significant capacity to represent the microenvironment of cancer cells and their associated mechanistic pathways at some point. In general, 3D-culture methods are based on three major systems a) 3D culture on scaffolds b) scaffold-free methods—organoids and spheroids.

#### 3.3.1. 3D Culture on Scaffolds 

They consist of a polymeric hard material-based support, where cells can first migrate then bind to a silk, collagen, laminin, alginate-based scaffold and fill the space among fibers to finally grow and divide [59]. This migration phase can result in porous and fibrous hydrogel, custom-rapid prototyping (RP), solid freeform (SFF) fabrication, microspheres, and native/ECM scaffolds types etc. On the one hand, scaffolds cultures are easily compatible with commercially available functional tests, DNA/RNA and protein isolation kits [60]. They are directly prepared for immunohistochemical analysis [61]. On the other hand, attached cells to scaffolds can flatten and spread like the ones cultured under adherent conditions [62]. Note that materials used to construct the scaffold may affect the adhesion, growth, and cell behaviors [63]. Scaling of scaffolds and topographic distribution of cells may present different behavioral attributes of cells. Hence, extraction and visualization of cells for analyses are sometimes confined to specific aspects [64]. Scaffolds types may vary in structure, function, shapes, and sizes; porous scaffolds are simple to make, but they lack connections between intercellular pores, reducing mechanical characteristics [54,65]. Fibrous scaffolds provide high nutrition and gaseous exchange properties across fibers, ensuring a stabilized and balanced setting [66]. Sometimes, fibrous scaffold formation is seen to be limited because of the small pore size restricting cellular integration with tissue host post implantation [67]. Whereas hydrogels are known to have elastic and flexible matrix maintaining well-standardized structural and functional matrix features [68]. In fact, this type is expensive to establish yet non adhesive cases require a secondary in vivo dressing support [69]. Recently it has also been noticed that the plastic used for cell growth also poses a problem due to nano-microplastic toxicity as explained by Singh et al. 2020 [70]. Inflammatory response occurrence is associated mainly with collagen, gelatin, alginate, and agarose polymers [71]. Controlled pharmacokinetic and compositional properties are checked in custom-rapid prototyping/solid-free form scaffolds but only a few polymer types can be used [20]. Moreover, large surface area scaffolds, known as microspheres, improve binding and growth criteria. Databases concerning associated adverse events are still limited thus complication due to reverse toxic effect needs further investigation [72]. Finally, native/extracellular matrix scaffolds achieve to mimic the in vivo microenvironment in terms of constituents, signals, and physiological properties as well as their restricted ability to fully control decellularization and cellular immunogenicity [73].

#### 3.3.2. Scaffold Free Methods—Organoids and Spheroids

Apart from scaffolds there are also scaffold-free methods for 3D-cell culture like organoids, another 3D model, is known to enable researchers identify the compounds targeting cancer cells through personalized medicine/drug screening-based subtype [74]. They impart healthy cancer tissues from the same patient [75,76], powerful drug response prediction during screening, detection of epigenetic variants linked to drug resistance [77,78], identification of protein mutation needed for treatment, individualization as well as exhaustive and extensive insight of intra-tumor heterogeneity [79], drug sensitivity of tumor subclones and toxicology assessment [80]. In some cases, failure to construct a homogenous environment can be related with the overgrowth of non-tumor components in tumor specimens [81,82]. Organoids can be used as tumorigenic organoids (tumoroids) based on assessing the role of mutational processes during tumorigenesis or microenvironment based on producing and releasing supporting factors for tumor survival [83,84]. Tumorigenic organoids ensure a better understanding of organ-specific mutagenic processes and model metastatic invasive mechanism [85,86,87,88]. While microenvironment organoids mimic tumor heterogeneity and cellular response based on tumor grade, stage, and treatment history, but in some cases this representation is incomplete due to the lack of fibroblasts, immune, and endothelial cells [89,90,91,92,93,94]. Currently, newly advanced microenvironment-based organoid models are being implemented. These new approaches are constantly proving their effectiveness and validity in terms of microenvironment, intrinsic factors expression, and physiological mechanisms [94,95] (Table 2 and Table 3).

Spheroidal 3D systems are also implemented among well-used and researched models. Multicellular tumor spheroids (MTS), a standard bi-dimensional culture of cancer cell lines showing a similar limited histological resemblance to the primary cancer are used of-late [110]. Thus, their clinical importance is determined by growth capacities as spherical colonies when suspended in cultures. Their availability and variability in sizes and shapes, cell clonal properties, easy handling, simple genetic maintenance, intensified metabolic and proliferative tumor gradients, providing constant multicellular chemo resistance resembling a true cancer patient case. This appropriateness serves as a potential tool for high-throughput drug testing [111]. In contrast, cell culture time, cell density, optimization process, and disability of all cell lines to form spheroids account for many underlying barriers [112]. Multicellular tumor-derived spheroids (MTDS), are similar to the (MTS) with additional specific tissue-related growth factors (TRGFs) and serum-free culture medium (SFCM) [113,114].

MTDS are enriched with cancer-stem cells (CSC) after CSC isolation and ex vivo expansion [115,116]. Cultures are confirmed by spheroids tumorigenicity, expression of CSC-related markers, pluripotency as well as investigation of CSC in vitro chemo resistance, to assess the intrinsic drug resistance in advanced cancers [117,118,119,120]. CSC regeneration is dependent on specific growth factors while serum addition can imbalance cellular differentiation and hence is avoided [121,122,123,124]. Heterotypic spheroids mimic the cellular heterogeneity of solid tumors and the resistance mediated by tumor-stromal cell interactions [125]. This model is composed of stromal cells as fibroblasts, immune cells, lymphatic endothelial cells, pericytes, and adipocyte [126]. The spheroid cells multiplied without creating agglomerates or making cell-to-cell connections. The capacity of a single cell to form a spheroid is thought to be suggestive of self-renewal and hence consistent with a CSC phenotype. The most appropriate method for extracting and assessing CSCs is to generate spheroids from a single cell on a microwell-based culture device [127]. The following cellular back up mediates drug-resistance mechanism in terms of angiogenesis, proliferation, invasion, and metastasis [127]. As well as it stimulates multiple pathways as DNA repair, proteasome activation, inflammation, ECM production, invasion and caspase [128,129]. Thus, integrating heterotypic spheroid in drug discovery studies [130,131,132,133,134].

### 3.4. Promising Single Cell Isolation Technique for Deriving Cancer Spheroids

In fact, the isolation of spheroid-forming cells is crucial for cancer stem cell (CSC) characteristics investigation. Till now, conventional tumor spheroid culture methods were not able to achieve a stable exemplary state because the aggregated spheroids potentially maintain their original heterogeneity and express various cells with multiple characteristics. In Jong Won Lee et al. study, isolated and enriched CSCs formed single cell-derived spheroids from gastric cancer cell lines. These obtained cells demonstrated higher self-renewal, enhanced stem cell markers’ expression and resistance to apoptosis compared with spheroid cells made by the traditional method. In another recent study, a single-cell RNA-sequencing of murine organotypic tumor spheroids undergoing programmed cell death 1 (PD-1) blockade was performed [135]. A discrete subpopulation of immunotherapy persisted cells (IPCs) that resisted CD8+ T-cell-mediated killing were obtained. In fact, these cells were able to express Snai1 and stem cell antigen 1 (Sca-1) along with hybrid epithelial-mesenchymal features characteristic of a stem-cell-like state, hence, demonstrating the power of high-resolution functional in vivo profiling [135]. So, cancer stem cells (CSCs) are proved to be tumor cells with initiating ability, self-renewal potential, and intrinsic resistance to conventional therapeutics [136]. Consequently, an effective isolation and featuring of CSCs is directly correlated with a more detailed investigation of tumorigenesis, heterogeneity, and chemoresistance. Moreover, a better understanding of CSCs will lead to novel era of both basic and clinical cancer research, reclassification of human tumors, and development of innovative therapeutic strategies in order to improve the probability of successful cancer treatment cases. Thus, single cell isolation technology is considered a novel approach representing a step forward in cancer stem cell studies.

### 3.5. Current Immuno-Oncology Effects of 3D Models

In vivo sites were considered in 3D hydrogels through many cellular cycles and proliferation mechanisms. Mainly, coordination between the stretch-activated channels (SACs), including TRPV4 and phosphatidylinositol 3-kinase (PI3K)/Akt pathway, stimulated cytoplasmic localization of the cell cycle inhibitor p27Kip1, hence allowing S phase entry and proliferation [70]. In polycystic kidney disease (PKD), human pluripotent stem cells and derived kidney organoids stimulated cystogenesis by ten-folds and cyclic adenosine monophosphate (cAMP) usage for a 1-cm cysts expansion in both PKD cases and organoids [137]. Cancer cell-migration process within a Matrigel-collagen hydrogel scaffolds was recently studied in H1299 lung cancer cells. It showed an enhanced β1 integrin expression and metalloproteinase activity, extracellular matrix-remodeling activity caused a matrix alignment and compaction intensifying the cellular tractions [138]. In the one hand, implantable scaffolds contributed to a better cancer immunotherapy effect, from a reduction in unresectable or incompletely resected tumors through T-cells proliferation [139]. In the other hand, elimination of solid tumors based on a systemic immune response was highlighted too [140]; enhancement in expansion, persistency, and antitumor efficiency of scaffold NK cells [141]; alleviation in the tumor-infiltrating MDSCs accompanied with an enhanced CD8+ T-cells release [142]; recruitment and activation of lymph nodes of DCs [143]. Consequently, injectable scaffolds provided a balanced packaging of CD11b+ CD11c+ DCs into the hydrogels [144]; collection and stimulation of immune cells [145]; regulation of the immunosuppressive tumor microenvironment activities [146]; activation of migratory DCs in tumor-draining lymph nodes combined with an induction of cytotoxic T-lymphocyte immune response [147]; increase in the percentage of CD8+ IFN-γ+ T cells [148]; as well as, the achievement of tumor suppression effects [149]; stimulation of powerful CD8+ IFN-γ+ T-cell immune response [150]; apoptotic induction of tumor cells and limitation of angiogenesis [151]. Achievement of powerful immune memory affected the resistance toward; secondary injection of tumor cells [152]. Collection of DCs, boosted the systemic TH1 and TH2 serum antibody and cytotoxic T cells [153]. Whereas BMDC activation marker expression and the innate immune cells infiltration were also performed [154,155,156]. Plus, recent research indicates that colorectal cancers include microbiota that differ from those found in a “normal” colon environment, and that these microbes might contribute to cancer growth [157].

### 3.6. 3D Co-Culture

Currently, 3D co-culture conceptualization presents the creation of a 3-dimensional (3D) tumor spheroid model capable of harboring and promoting the growth of anaerobic bacteria. Bacteria-tumor cell interactions and metabolic crosstalk were widely examined using bacterial growth kinetics, cell morphology and lysis, cancer-related gene expression, and metabolomics [158]. This bacterium-spheroid co-culture model allows for mechanistic analysis of the role of anaerobic bacteria in tumor microenvironment description of a 3D tumor spheroid model co-cultured with cancer-relevant, endogenously found anaerobic bacteria. Bacteria-spheroid co-cultures (BSCCs) have previously been described in investigations with genetically tractable anaerobic bacteria as possible gene-delivery sources for therapeutic applications [68].

## 4. Drug Discovery

### 4.1. Drug-Dose Response

As cancer treatment options are constantly and gradually expanding, chemotherapy failure and disease recurrence are still the most dominant case scenarios. Cancer cells are complex in nature, suggesting critical in vivo cellular responses and mechanisms. As mentioned previously, 2D-cell culture studies are the main assets to discover cancer cells activity. Recently, 3D-cell lines added a clearer in vitro reflection of drug activation or inhibition mechanism of action in a cancer cell. Transcriptomic and proteomic trials proved that the human choroid plexus ChP organoids barrier’s selectivity to small molecules was similar to the in vivo setting, in addition to the predictor ability of ChP-CSF organoids related to CNS permeability of novel compounds [159]. While analysis of spheroidal colon cancer cells showed a diminished activity of AKT, mammalian target of rapamycin (mTOR) and S6K signaling pathways limit its physiologic ability to closely coordinate the tumor areas around vessels in vivo [160]. A comparative evaluation of antineoplastic efficacy of drugs paclitaxel and docetaxel in terms of cytotoxicity, cell proliferation, and gene expression assays between 2D and magnetic 3D cultures was studied. Lower cell proliferation rate, more resistance to paclitaxel and docetaxel, and altered gene-expression profile was shown in 3D-cell culture compared to its 2D counterpart [161]. These findings, suggested the classification of 3D-cell lines as a promising option for a better understanding of cancer resistance mechanisms toward chemotherapeutic agents as well as formulation and discovery of novel agents. The prominent value of 3D spheroids as an early phase drug screeners toward a personalized treatment for Uveal melanoma (UM) patients and highly standardized drug testing was studied. These (UM) tested cell lines produced spheroids genetically identical to the original sampled tumor of varying sizes and compactness. Consequently, in vitro drug assays revealed doxorubicin’s crossing potential in the spheroid core while selumetinib affected largely the peripheral cells [162]. Drug-resistance studies have also been carried out in head and neck squamous cell carcinoma (HNSCC) tumors which are known to be majorly unresponsive to therapies. So, cultured (HNSCC) tumor cells in a 3D-spheroidal environment released high levels of CDH1, NANOG, and SOX2 compared to limited concentrations in 2D. 3D-grown HNSCC cells showed decreased sensitivity to cisplatin and cetuximab (antiEGFR) treatment mimicking the physiological setting and approaching the true tumor behaviors [162]. Proliferation, genetic expression, and chemoresistance of prostate tumor cell lines, PC3, LNCaP, and DU145 comparison within 2D and 3D environments were also assessed. Post receiving paclitaxel and docetaxel, a lower cell proliferation rate, more resistance to paclitaxel and docetaxel, and altered gene expression profile were shown in 3D-cell culture compared to its 2D counterpart [163]. Porous scaffolds setting reflected the actual in vivo slow and sustained release of cisplatin followed by fibroblast cells adherence and proliferation during cancer chemotherapeutic treatment [164]. Similarly, 3D-cultured breast cancer cells on the decellularized scaffolds showed also reduced sensitivity to doxorubicin in comparison to 2D-cell culture [165]. 3D-spheroidal breast cancer cells increased cell–cell contact, matched cell morphology characteristics with the in vivo tumors. Importantly, these cells showed an increase in resistance to dacarbazine and cisplatin [166]. In fact, multiple studies valorized the contributive application of 3D-culture models, mainly spheroids in the evaluation of chemotherapeutic agents’ resistance in ovarian cancer as well as the development of new techniques for assessing the treatment sensitivity. Xu et al. highlighted the important role of E-cadherin in spheroid formation and drug resistance to cisplatin [167]. Raghavan et al. formulated a novel 384-well hanging drop tumor spheroid for the purpose of testing sensitivity to cisplatin chemotherapy [168]. As well as, a patient-derived 3D hanging drop spheroid platform with ALDH+, CD133+ ovarian cancer cells was used to screen the effects of chemotherapy drugs [169]. Hence, Aihara et al. developed a novel 3D-cell culture technique using FP001 polymer for anticancer agents screening and which also facilitated the homogenous spheroid culture [170]. Some research also the focused on specific biomarkers’ effect on the efficacy of ovarian cancer treatment. Yang et al. explored the role of bcl-2 in response to platinum drugs used in treatment of ovarian cancer [171]. Recent advanced clinical trials by Rashidi et al. developed an in vitro 3D model to study the stemness and chemoresistance in ovarian cancer. This obtained spheroid technique is characterized by cellular enrichment with stem cell markers and emergence of platinum-resistance phenotype [172]. Whereas Shuford et al. developed an ex vivo-patient-derived 3D spheroid model for drug testing which succeeded to link clinical response to therapy and in vitro response in some patients [173]. Three-dimensional models are recognized as a potential bridge between in vitro monolayer cultures and in vivo animal testing. This is due to the fact that 2D models produce deceptive findings with low predictive value for clinical efficacy, because monolayers frequently fail to match the actual state in tumors, many medicines fail in clinical trials. Compared to monolayer cultures, almost all anticancer medicines are less efficient in a multicellular spheroid model. The changed treatment response of spheroids relative to 2D culture is thought to be the result of three significant changes: first, increased resistance combined with lower drug diffusion; second, the effects of the changed cellular environment; and third, the small proportion of proliferating cells that are selectively targeted by cytotoxic medicines [174]. Spheroid studies have a greater predictive value for cytotoxicity and therapeutic efficiency than monolayer cultures. Furthermore, 3D co-cultures of diverse cancer cells, including pancreatic cancer cells, are more resistant to a wide range of chemicals than 3D cultures of only one kind of cell. For example, typical PDAC therapies such as gemcitabine and oxaliplatin require 200-fold greater doses to achieve the same IC50 value in spheroids as in monolayer cultures. As a result of this phenomena, it has been suggested that therapies be tested not only on 3D models but also on 3D co-culture models in order to discover substances that are beneficial in both experimental setups. This reduces the cost of treatment testing since unsuitable chemicals are eliminated prior to animal testing. Positive selection of chemicals happens in addition to the so-called negative selection, which refers to the rejection of inefficient compounds. Some targets or pathways are increased in the 3D environment and hence make suitable therapeutic targets. Positive selection is demonstrated by the PI3K inhibitor wortmannin and the wortmannin analogue PX-866, which were unsuccessful in monolayer culture but inhibited spheroid formation in glioblastoma, prostate, breast, and colon cancer cell lines. Importantly, spheroid inhibition coincided with the outcomes in human tumor xenografts. Similarly, in spheroid ovarian and prostate cultures, the proteasome inhibitor PS-341 showed equivalent or greater inhibitory potential. The list of medications with positive selection has been extended elsewhere [175].

### 4.2. Microfluidics-Organs-on-Chips

Microfluidics-Organs-on-chips: Organs-on-chips are biomimetic systems that imitate the microstructures, dynamic mechanical characteristics, and biochemical capabilities of biological organs. The advancement of microfluidic technologies have enabled the precise control of the microenvironmental factors (microfluidic), which resulted in long-term and regulated 3D-cell culture models, produced by utilizing biocompatible microfluidic chips that allowed tissue manipulation [174]. Organs-on-chips have transformed 3D-cell culturing by enhancing existing methods and introducing new possibilities. Microstructures composed of collagen or polymer-based membrane are created within the chip’s micro-channels to better imitate the organization and functioning of real tissue. In contrast to typical 3D-cell culture, a human breathing lung-on-a-chip is a model of the alveolar-capillary. It incorporates a flexible polymer membrane that allows movement similar to that of a real human lung. Manipulation of small doses of fluids in micro-channels facilitated by microfluidic enabled organs-on-chips to have fine flow control on various scales to “irrigate” the cell growth. As a result, it is feasible to add items that are required by the cells. Organs-on-chips can also aid in the development of a fragmented microfluidic system that allows for controlled co-culture and the reconstitution of a tissue–tissue interface [176]. As a result, many disease models types in the case of malignant breast and brain tumors, or the behavior of breast cancer cells when they become invasive carcinoma, can be created. This innovative technique is ideal for the demanding and complicated needs of 3D-cell growth. Indeed, it aids in simulating tissue interfaces to imitate organ function while meticulously monitoring and controlling events. The micro-channels, which house a three-dimensional cell culture, are linked to holes through which fluids passing through are combined. Those routes are precisely controlled by microfluidic output devices (flow monitoring and control systems) that are governed by a microfluidic chip, and the controlled cell development inside micro-channels is directed by a suitable substrate with sufficient mechanical, chemical, and surface characteristics. At the end of the day, organs-on-chips produce well-organized tissue and its components [177]. 3D-cell culture models are progressively being acknowledged as the most biofidelic in vitro representations of tissues for study. Biomatrices and bulk populations of cells taken from tissues or cell lines are used to create these models. We provide an alternative approach for cultivation of individual cells in relative isolation from the rest of the population under physiologically relevant matrix conditions. Matrix gel islands are placed on a cell culture plate to serve as a platform for receiving and growing individual single cells; a glass capillary-based microfluidic system is utilized to extract each required single cell from a population and seed it on top of an island. Using breast and colorectal cancer as examples, individual cells grow into tumors or parts of tumors with varying features of the initial cancer type and aggressiveness, as demonstrated [178].

With the advancement of rapid cell culture system and its associated plastic toxicity the advent of artificial intelligence and machine learning-based approaches can revolutionize this system [179]. In vitro two-dimensional (2D) cancer cell growth does not replicate the three-dimensional (3D) architecture, heterogeneity, and complexity of human tumors. PREDECT (www.predect.eu accessed on 1 July 2021), an Innovative Medicines Initiative (IMI) collaboration on january 2016, described in vitro models of three solid tumor types with the objective of capturing features of tumor complexity and heterogeneity.

### 4.3. Intrinsic Pathways/Genetic Factors’ Expression

Gene-based technologies are exponentially implemented as an additional tool for unmasking any underlying pathogenetic effect of human genetically based disease-causing mutations. As we already discussed, cancer is one of the major conditions underlying genes-associated polymorphisms. Thus, multiple clinical trials were recently implemented. 3D neural differentiation of stem cells was compared to multiple primary brain tissue samples. In vitro, organoids intrinsically achieved chromatin state transitions closely related to the in vivo human forebrain development [180]. Similarly, organoid cells’ evolution through symmetrical spheres originates from a transient activation of the transcriptional regulator YAP1, resulting in the activation of Notch and DLL1 and stimulating the symmetry-breaking event and the first Paneth cell formation [181]. Metabolic functions were also highlighted based on Mimcd3 cells cultured in 3D spheroids for 48 h matching the normal metabolic functions as the in vivo cells isolated from nephrons, specifically the decrease in tricarboxylic acid cycle and glycolysis intermediates. Whereas, the elevation in betaine, taurine, and 1, 24-25-trihydroxyvitamin D produced and the suppression of the pentose-phosphate-pathway mismatched 2D cells secreted levels [182]. Ishiguro et al. revealed the inhibitory effect of Rho kinase ROCK inhibition in ovarian cancer as a promoter to ovarian cancer stem cell (CSC) proliferation and malignant progression [183]. Chen et al. and Lu et al. described the importance of regulating putative stem-like cell markers and formation of epithelial ovarian cancer spheroids through the activation of STAT3 [184,185]. Boylan et al. suggested cell adhesion molecule Nectin-4 as a main element of ovarian cancer spheroid formation [186,187]. Senkowski et al. showed gene expression analysis of 3D multicellular tumor spheroids against 2D monolayer cells in another study. The changes in gene expression were discovered to be the overexpression of genes involved in hypoxia response and the downregulation of genes involved in cell cycle progression [188]. Furthermore, during oxidative phosphorylation inhibition, the mevalonate pathway was elevated in quiescent cells of 3D spheroids, which was connected with quiescent spheroids’ viability deficit when treated with oxidative phosphorylation inhibitors and mevalonate system inhibitors. This showed that anticancer treatment responses of 3D tumor spheroids were context dependent. The genomes of 3D glioblastoma multiforme (GBM) cells cultured on polylactic acid porous scaffolds were recently compared to the genomes of GBM cells cultured in 2D-cell culture settings. When compared to the 2D-cell growth conditions, the 14-day 3D GBM cells activated 8117 genes and downregulated 3060 genes [189]. Pathway analysis in the Kyoto Encyclopedia of Genes and Genomes revealed that genes associated with the PPAR and PI3K-Akt signaling pathways were mostly upregulated, whereas genes involved in metabolism, ECM receptors, and the transforming growth factor pathway were mostly downregulated. The in vitro 3D tumor data would be valuable information for a better understanding of both intrinsic and extrinsic factors. A 3D-tumor model of this type has the potential to be used as a platform for anti-GBM medication screening [190].

## 5. Next-Generation Therapies

SpheroidPicker is an artificial intelligence-enabled Low-Cost 3D Cell Culture Delivery System presented as an Automated 3D Cell Culture Manipulator Robot Using Deep Learning. It is made up of a light microscope, a micromanipulator, a syringe pump, and a computer controller. The method analyses morphology-based features on spheroids and transfers the most relevant ones between different samples. It can pick samples from typical sample holders, such as Petri dishes and microwell plates, and then transfer them to a range of holders ranging from Petri dishes to 384 well plates. The apparatus is capable of semi-automatic and completely automated spheroid transfer. This produces highly controlled experimental settings and removes non-trivial side effects of sample variability, which is an important element of next-generation precision medicine [191]. Advances in 3D-cell culture, tissue engineering, and microfluidics have resulted in the creation of “cancer-on-a-chip” systems, which increase the capacity to mimic the TME in vitro and enable high-throughput analysis. Advances in the creation of cancer-on-a-chip systems, their implications for drug discovery, the difficulty of utilizing this technology for improved cancer therapy, and potential integration with artificial intelligence for enhanced predictive drug-screening models have been carried out [192]. Increasingly complicated 2D and 3D mono- and stromal co-cultures, as well as precision-cut tumor slice models, were created. Robust methods are presented for the creation of these systems. Tissue microarrays were made from all of the animals, allowing for immunohistochemical investigation of individual cells and the capture of heterogeneity [193]. Image analysis was also used to characterize 3D cultures. Detailed step-by-step protocols, representative datasets from the 2D, 3D, and slice models, as well as enhanced analytical approaches, have been developed and are given. Carcinomas, often known as solid tumors, contain a complex microenvironment, a diverse cellular population, and a three-dimensional (3D) architecture [194].

## 6. Applications of 3D-Cell Culture in Translational Oncology and Precision Medicine

Translational oncology constitutes the main bridging factor between basic research and clinical practice. Today, translational oncology medicine benefits from the vast availability of information resulting from newly developed 3D models. In fact, the main updated feature of translational oncology is highlighted through Bioprinting which allows for the creation of very complex 3D structures using live cells. This cutting-edge approach has grown in popularity and application in a variety of disciplines. Bioprinting techniques have been created to design live cells, biological macromolecules, and biomaterials in an efficient and timely manner. These technologies have a lot of promise for use in cancer research. Bioprinted cancer models outperform prior 2D models by simulating 3D complexity and allowing for physiologically realistic cell–cell and cell–matrix interactions. Bioprinting methods are based on inkjet, microextrusion, and laser technologies, and 3D cancer models are compared to 2D-cancer models. We address bioprinted models that replicate the tumor microenvironment, allowing for a better knowledge of cancer pathophysiology, anticancer drug screening, and cancer therapy development [195]. To change health care, 3D printing in the medical area and design must think beyond the box. The three major foundations of this new technology are the capacity to treat more people where it was previously not possible, the ability to get outcomes for patients, and the ability to spend less time in the direct case of medical professionals. In a nutshell, 3D printing allows doctors to “serve more patients without compromising results.” 3D printing allows us to create the drug in a specific shape, making medicine more appealing to children. It is critical to understand that altering the form of a capsule does not have to result in a change in dosage or pharmacological characteristics such as drug release or disintegration rate. As a result, 3D printing, like any new technology, has offered several advantages and potential in the medical area. Each individual situation in which 3D printing has found use in research analysis exemplifies this. However, in order to ensure its proper usage, it must be supported by up-to-date and relevant laws [196].

## 7. Conclusions

As we have already mentioned, the ability of 3D-cell culture cells to reflect the in vivo tumor architecture as well as to be a major tool in anticancer drug sensitivity testing classified them as the most promising experimental models in preclinical research. However, still an official FDA approval for an ideal model (either 2D- or 3D-cell culture based) is not yet in hands. In fact, continuous challenges in terms of tumor heterogeneity, metastasis, invasion, therapy-resistance, and tumor relapse encountered are the crucial assets for developing and enhancing the usage of 3D-culture cells to achieve a successful cancer treatment and a good patient prognosis. Cell culture is an essential step in drug development, cancer research, and stem cell research [197]. Most cells are now cultivated in two dimensions (2D), however new and enhanced approaches that employ three-dimensional (3D)-cell culturing techniques provide persuasive evidence that considerably more sophisticated studies may be undertaken, generating important insights. The cell environment may be adjusted in 3D-cell culture procedures to imitate that of a cell in vivo and offer more precise data regarding cell-to-cell interactions, tumor features, drug discovery, metabolic profiling, stem cell research, and other sorts of disorders [198]. Scaffold-based approaches such as hydrogel-based support, polymeric hard material-based support, hydrophilic glass fiber, and organoids are used, each with its own set of benefits and uses. Similarly, scaffold-free approaches such as hanging drop microplates, magnetic levitation, and spheroid microplates with ultra-low attachment coating are employed. Through the use of organoids, 3D-cell culture has the potential to give new techniques to research organ behavior, and it is projected to eventually bridge the gap between 2D-cell culture and animal models. The current study compares 2D-cell culture and 3D-cell culture, gives information on the various 3D-culture techniques, and focuses on the current and future applications of 3D-cell culture. Methods for advancing research are provided by both 2D- and 3D-cell culture techniques. 3D-cell culture, on the other hand, has demonstrated the ability to totally transform the way novel drug therapies are tested, illnesses are modelled, stem cells are used, and organs are transplanted. As 3D-cell culture becomes more prevalent, the procedures will get more refined, and more complex approaches will emerge. The latest being 3D bioprinting of culture scaffolds [199]. Researchers that are currently using 2D-cell culture models to evaluate novel pharmacological treatments should seriously investigate 3D-cell culture possibilities. The advantages of co-culture cells in 3D are better to those of 2D-cell culturing, and as tissue engineering techniques develop, tumor models, cancer treatments, and disease testing methods will all improve. Through this review, we delved more into the realistic three-dimensional reflection of the physiological microenvironment in terms of cellular interactions and differentiation as well as exchange mechanisms. At some point, 3D-cell lines characteristics achieved an authenticated demonstration matching common cell behaviors and is associated with promising evaluation of other mechanistic approaches. Moreover, 3D concept illustrated drug resistance as one of the major assets behind cancer cells survival potential and ability to multiply easily in a shorter time span. Thus, healthcare professionals are at a closer stage of studying, discovering, and formulating novel anticancer drugs, highly efficacious with a lower risk of adverse events and complications.

## Figures and Tables

**Table 1:** Comparative assessment of 2D- and 3D-cell culture properties.

Main Properties	2D-Cell Culture	3D-Cell Culture	References
Cell morphology	Cells grow as a flat and elongated 2 dimensional monolayer on petri plates or flasks	Cells grow as 3D spheroids within multiple layers	[37]
Cell microenvironment	Equal quantity of nutrients and growth factors received by all cells Majority of cells are in the same cell cycle Absence of in-vivo like environment	Variable quantity of nutrients are ingested by cells based on the cell cycle Mimic an in-vivo cell mechanism	[38,39]
Cell interaction	Coordination between cells is limited	Cell–cell interaction is active	[40]
Cell differentiation	Deficient	Efficient	[41]
Cell proliferation	Irregularly rapid intensity	Gradual and balanced	[42]
Drug response	Cellular resistance is not well expressed Drug pharmacodynamic /pharmacokinetic mechanisms are not well studied	Drug- dose response is well studied as it is more likely to matches the in vivo resistance	[17,43]
Molecular mechanisms	Variable gene/protein expression, mRNA splicing, and cells biochemistry	Expressed genes, proteins, mRNA, and other cells processes are well assessed and measured	[15]
Cost	Commercially available, cheaper than 3D culture	Fewer commercially available, more expensive than 2D culture	[44]
Testing and interpretation	Easily assessed Better for extended culturing	Needs a longer assessment time Difficult to interpret data	[45]
Apoptosis	Easily subject to apoptosis by drugs	Drugs are subject to higher resistance rates	[42]
Response to extrinsic stimuli	Not a valid reflection of the biological cell response Unable to respond to gravity since their inability to evolve into a 3D cell	Effective reflection of the intrinsic response Constantly able to respond to gravity since they can formulate their 3D cells	[46,47]

**Table 2:** Newly advanced microenvironment-based organoid models.

	Cancer Organoids Type I	Cancer Organoids Type II	References
Culture setting	Cultured directly from tumors preserving endogenous immune cells and other non-epithelial cell types	Co-cultured with immune cells isolated and separately expanded	[96,97]
Approach type	Holistic	Reductionist	[98,99]
Validity	Both are equally valid in terms of:		
	Cell–cell interactions		[100,101]
	Modeling immune checkpoint blockade		
	Testing CAR-T-cell-mediated cytotoxicity		
	Highly informative platform for the development of cancer immunotherapy		
Recent trials	Findings in both original tumors and organoids:		
	Conservation of T-cell receptor repertoire		[102]
	PD-1/PD-L1 immune checkpoint axis functional		[102]
	High level of differentiation into alpha-SMA and inflammatory mediators such as IL-6		[102]
	Supported tumor growth and formation of niche enriched Wnt-ligands		[103]
	Model diabetic vasculopathy needed for a well perfused system compromising arterioles and venules		[104,105]

**Table 3:** New approach derived from organoids.

	Organ-on-a-Chip Technology	References
Model type	Microfabricated cell culture device	[106]
Function	Reproduction of key functional features of human organs in vitro, mainly	
	Cytostructural organization of different cell types	[106]
	Dynamics of flow perfusion	
Advantages	Each “organ functional unit” can be interconnected based on microfluidic channels simulating multi-organ interactions	[107]
	A reflective model for metastatic process study	
Recent trials	Combining both organoids and organ-on-a chip models thus taking the best features of both systems:	
	Creation of a 3D-perfusable blood vessel network capable of delivering nutrients and/or drugs to patient-derived breast cancer	[108]
	Evaluation of Bleomycin cytotoxicity through formulation of a multi-organ on a chip system composed of liver and heart organoids perfused in a closed loop with a micro-engineered lung tissue	[108,109]
	Study of the metastatic spread of primary lung cancer through lung-brain-liver-bone-on-a-chip model	[109]

## Data Availability

Not applicable.
